# Bioactive Compounds and Volatile Profiles of Five Transylvanian Wild Edible Mushrooms

**DOI:** 10.3390/molecules23123272

**Published:** 2018-12-11

**Authors:** Melinda Fogarasi, Sonia Ancuţa Socaci, Francisc Vasile Dulf, Zorița Maria Diaconeasa, Anca Corina Fărcaș, Maria Tofană, Cristina Anamaria Semeniuc

**Affiliations:** 1 Department of Food Engineering, University of Agricultural Sciences and Veterinary Medicine of Cluj-Napoca, Cluj-Napoca, Calea Mănăştur 3-5, 400372 Cluj-Napoca, Romania; melinda.nagy@usamvcluj.ro (M.F.); cristina.semeniuc@usamvcluj.ro (C.A.S.); 2 Department of Food Science, University of Agricultural Sciences and Veterinary Medicine of Cluj-Napoca, Calea Mănăştur 3-5, 400372 Cluj-Napoca, Romania; zorita.diaconeasa@gmail.com (Z.M.D.); anca.farcas@usamvcluj.ro (A.C.F.); maria.tofana@usamvcluj.ro (M.T.); 3 Department of Environmental and Plant Protection, University of Agricultural Sciences and Veterinary, Medicine Cluj-Napoca, Calea Mănăştur 3-5, 400372 Cluj-Napoca, Romania; francisc.dulf@usamvcluj.ro

**Keywords:** mushrooms, chemical compounds, fatty acid, volatile profile, phenolic compounds

## Abstract

This study aimed to determine the chemical composition, fatty acids, volatile profile and phenolic compounds profiles from five wild edible mushrooms (*Agaricus bisporus, Pleurotus ostreatus, Cantharellus cibarius, Boletus edulis, Lactarius piperatus*) from Romania. The results indicated that the dried fruiting bodies of selected mushrooms were rich in proteins (36.24 g/100 g dw-*Boletus edulis*) and carbohydrates (62.45 g/100 g dw-*Lactarius piperatus*). 4-Hydroxybenzoic acid and cinnamic acid, were the main phenolic compound present in all selected species. Additionally, the fatty acid pattern included polyunsaturated acids in more than 60% of all fatty acids followed by monounsaturated fatty acids (30%). For the studied mushroom samples, the main volatile compounds identified by the gas chromatography-mass spectrometry were hexanal, benzaldehyde and dodecanoic acid. According to the obtained results, the fruiting bodies of selected Romanian mushrooms are a rich source of bioactive molecules indicating that they may be further exploited as functional ingredients in the composition of innovative food products.

## 1. Introduction

Over the last decade, the proven health-promoting abilities of different food classes, especially foods originated from unpolluted areas (i.e., mountains) gain the attention of consumers and food industry. It is well known that, mushrooms are consumed as a delicacy for their texture and flavour and have an important nutritional value due to their high protein, essential amino acids and fibres content complemented by a low fat content [1]. Even though mushrooms do not constitute a significant portion of the human diet, their consumption continues to increase mainly due to their functional benefits attributed to the presence of bioactive compounds which may act as antioxidants, anticancer and antimicrobial agents [2].

Simultaneously, edible mushrooms are regarded as an important dietary supplement for people interested in calorie restriction because of the low amount of fat, cholesterol and high concentration of fibre. It is well known that many cultures and civilizations around the world, especially from South and Central-Eastern Europe use wild edible mushrooms in cooking, traditional medicine, and other anthropogenic applications, this tendency being increasingly more accepted [3].

Moreover, mushrooms’ biological activities have been reported in the scientific literature, including antitumor and immunomodulating effects, inhibition of platelet aggregation, reduction of blood cholesterol concentrations, prevention or alleviation of heart disease and reduction of blood glucose levels, antimicrobial activity and significant antioxidant capacity [4]. Also, dried mushrooms were used as feed in poultry nutrition with positive in vivo antiprotozoal and antioxidant effects [5,6]. Therefore, mushrooms can be used both as a functional food or ingredient in functional products.

The mountainous area of Romania, due to climatic conditions and flora diversity, is one of the European regions with high wild edible mushrooms diversity. Despite of their great popularity, data regarding the chemical composition and bioactive compounds of the wild mushrooms available in the region as well as their nutritional value are very scarce. Based on the above discussions, the aim of the present study was to provide a comprehensive study on the chemical and nutraceutical composition of five selected mushrooms species (*Agaricus bisporus, Pleurotus ostreatus, Cantharellus cibarius, Boletus edulis, Lactarius piperatus*) collected from Romania as well as on some of their biological activities. The selection of the above mushroom species was based on their popularity and also because they are the most harvested wild edible mushrooms in Romania.

## 2. Results and Discussions

### 2.1. Proximate Composition

Research on the chemical composition of the selected mushrooms was conducted to target different classes of bioactive compounds. To the best of our knowledge, there are only a few studies referring to the chemical composition of mushrooms fruiting bodies originating from Romania [3]. 

Results concerning the nutritional value are presented in Table 1. Carbohydrates were the most abundant macronutrients (43.01 ± 0.70 − 65.06 ± 0.11g/100 g dw) followed by proteins (17.92 ± 0.18 − 36.24 ± 0.12 g/100 g dw) and fats (1.26 ± 0.08 − 2.69 ± 0.05 g/100 g dw).

In this study, the highest protein content was found for *B. edulis* (36.24 g/100 g) followed by *A. bisporus* (34.68 g/100 g). Samples of *C. cibarius* and *P. ostreatus* had lower protein content (21.03 g/100 g and 17.92 g/100 g respectively), in agreement with the results of Beluhan et al. [7]. Nonetheless, Kalac et al., in a review regarding the European wild growing mushrooms, reported a wide range in protein content (17.2–59.4 g/100 g dw) for the mushroom species [7,8]. These differences may be attributed to a number of factors that can influenced the mushrooms protein contents, namely the type of mushroom, the stage of development, the part sampled, level of nitrogen available and the harvest location [9]. Moreover, for the same mushroom species, the chemical composition may be influenced by the pedo-climatic conditions.

Generally, mushrooms are low-calorie foods, but their energy value is not exclusively due to their fat content but also to the other macronutrients (see the energy calculation formula, Section 3.2.). Thus, it is possible for a mushroom species with a higher fat content to have a smaller energy value than a mushroom species with a lower fat content (e.g. *B. edilus vs L. piperatus* in Table 1). Similar results were obtained by Heleno et al. when they determined the proximate composition of two wild edible mushrooms from Portugal [10].

The ash content of analyzed samples also varied among species, with *P. osteoratus* and *C. cibarius* (11.11 and 9.57 g/100 g respectively) being the richest ones while *B. edulis, L. piperatus* and *A. bisporus* had lower values (8.38, 8.30 and 7.24 g/100 g respectively).

The carbohydrate contents of investigated wild edible mushrooms were between 43.01 g/100 g and 65.06 g/100 g. Carbohydrates calculated discounting protein, ash and fat levels, were the most abundant macronutrients and the highest levels were found in *C. cibarius* (65.06 g/100 g). Carbohydrates content includes also fibre such as the structural polysaccharides β-glucans, chitin, hemicelluloses and pectic substances.

For the evaluation of the nutritional value of mushrooms, the most important factor to be considered is their dry weight content. It is known that the dry weight content of fresh mushrooms is generally 5–15% and the nutritional profiles of mushrooms are directly affected by their moisture content [11]. Moreover, the moisture content of mushrooms depends on the harvesting time, maturation period and pedo-climatic conditions [7]. In our study, the proximate composition was determined for the dried mushroom samples, thus the lower moisture content of 2.14–14.20 g/100 g dw. Taken into consideration that all the samples were dried in the same conditions (at 25 °C for 24 h), the differences noticed between the obtained moisture contents of mushroom powders may be attributed to the differences in the size of fruitting bodies but also to the initial water content.

The proximate composition and the nutritional value of the selected mushrooms were similar to those of other European species of wild growing mushrooms, where carbohydrates and proteins were also described as being the most abundant macronutrients [7,8].

### 2.2. Fatty Acid Composition

The result for fatty acid composition, total saturated fatty acid (SFA), monounsaturated fatty acids (MUFA), and polyunsaturated fatty acids (PUFA) of the studied mushrooms are shown in Table 2. 

**Figure 1:**
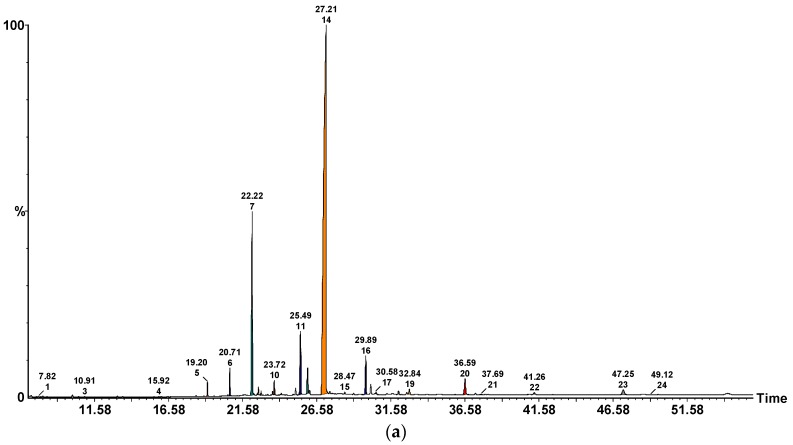

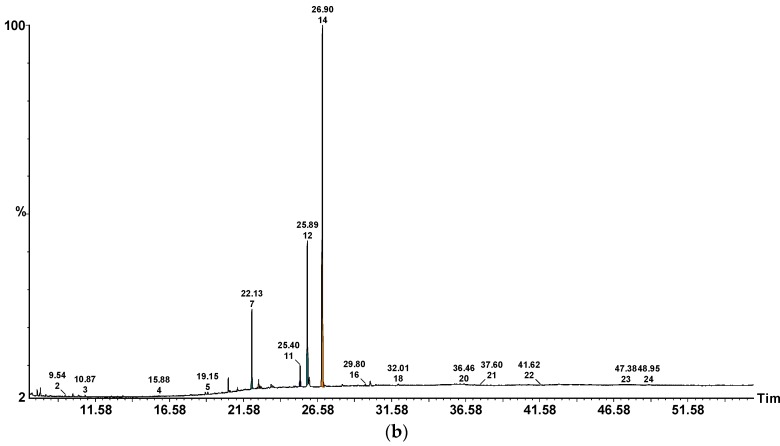
GC-MS chromatogram and retention time of fatty acids methyl esters (FAMEs) in the TLs of dried mushroom sample: (**a**) *A. bisporus*; (**b**) *B. edulis*. The numbering of the peaks refers to Table 2.

In general, the major fatty acid found in the studied samples was linoleic acid (C18:2n-6), followed by oleic acid (C18:1n-9) and palmitic acid (C16:0). In fact, it is known that linoleic acid is the precursor of 1-octen-3-ol, known as the alcohol of fungi, which is the principal aromatic compound in most fungi and might contribute to mushrooms flavor [12]. Besides the 3 main fatty acids already described, 20 more were identified and quantified. PUFA were the main group of fatty acids in all species excepting *C. cibarius*, in which SFA were predominat. Considering total MUFA content, *A. bisporus* (2.55%) had the lowest value but contained the highest PUFA content (71.75%), due to the higher contribution of linoleic acid. Thus, the analyzed mushroom samples could be considered excellent sources of linoleic acid which in human body is the precursor of other long-chain n-3 and n-6 fatty acids. A typical chromatogram for the fatty acids profile of *A. bisporus* and *B. edulis* are presented in Figure 1.

### 2.3. ITEX/GC-MS Profile of Volatile Compounds 

Each mushroom variety can be differentiated from others based on its aroma which is strongly influenced by the volatile and semi-volatile constituents. The volatile profile of the selected five wild Transylvanian mushrooms was assessed by headspace in-tube extraction coupled with gas-chromatography-mass spectrometry (HS-ITEX/GC-MS). A number of 34 compounds were separated and tentatively identified, their relative concentrations and characteristic odor being presented in Table 3.

The volatile compounds from mushroom samples could be divided in several functional groups: alcohols, aldehydes, ketones, terpenoids, acids, sulphur compounds and others. As it can be noticed, the most abundant were the aldehydes and ketones as well as the sulphur compounds in the case of *B. edulis* and *L. piperatus*. Aldehydes and ketones were also the dominant compounds in the mushrooms varieties analyzed by Zhang et al. [13]. The main aldehydes present in all samples were 2-methyl-pentanal, hexanal and benzaldehyde but their concentration varied greatly depending on the mushroom variety. 

The alcohols are considered to contribute to the “mushroom-like” aroma. *B.edulis* and *C. cibarius* have the highest concentration in alcohols, especially in 1-octen-3-ol. Beside the mushroom variety, the content in 1-octen-3-ol may be influenced by the mushroom maturity stage, by the storage time, by the technique used for the extraction of volatile compounds [13,14], but it may also be lost during the drying process. Thus, for example, in straw mushrooms, 1-octen-3-ol concentration decreases gradually during maturity while the content in 3-octanone increases. In other species, like oyster mushrooms (*P. ostreatus*), the amount of these compounds remains relatively constant during maturation [13]. *B.edulis* and *C. cibarius* are the richest in 1-octen-3-one and 3-octanone which also impart a mushroom-like aroma. 

The *L. piperatus* variety is characterized by the high amount of sulphur compounds (51.39%), while in *P.ostreatus* and *A. bisporus* these compounds are absent. Sulphur compounds were also identified by Leffingwell and Alford [14] in *Calvatia gigantea* variety, but only in trace amounts.

### 2.4. Phenolic Content

Flavonids and phenolic acids identification and quantification from selected mushrooms, commonly used in food consuming/industry were done by high-performance liquid chromatography coupled with mass spectrometry (HPLC-MS). The compounds identification was achieved based on their retention times, their UV-Vis absorption spectra and mass spectra of analyzed samples and also by comparison with available literature data [15,16] (Table 4). 

**Figure 2:**
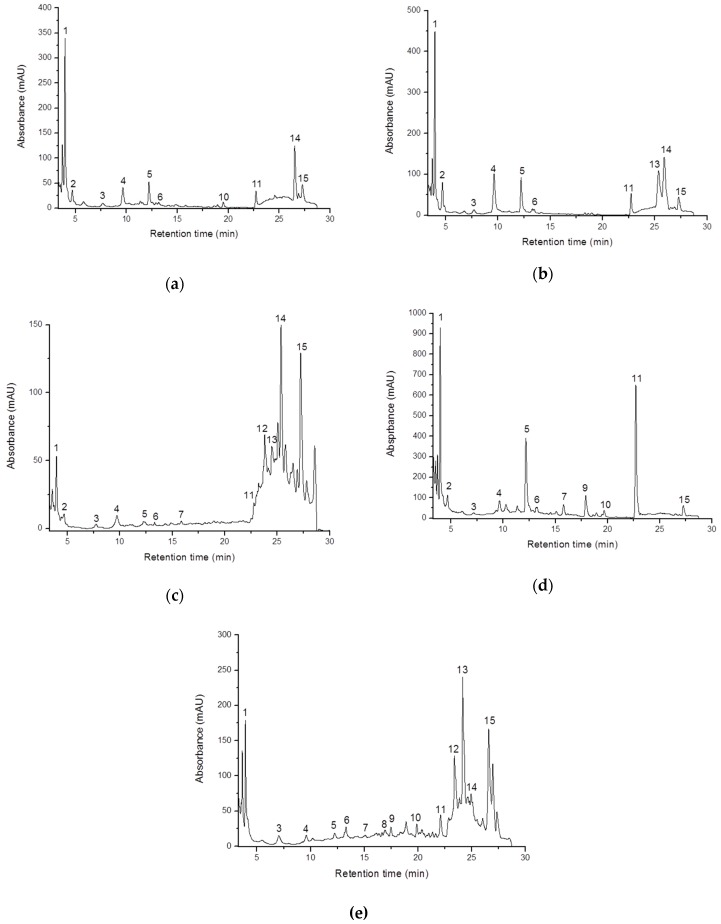
HPLC chromatograms of mushrooms extracts recorded at 280 nm: (**a**) *Pleurotus ostreatus*, (**b**) *Agaricus bisporus*, (**c**) *Cantharellus cibarius*, (**d**) *Boletus edulis*, (**e**) *Lactarius piperatus*. The numbering of the peaks refers to Table 4.

There were in total 15 identified compounds, their chromatograms being shown in Figure 2. Overall, 4-hydroxybenzoic acid, cinnamic acid and 5-feruloylquinic acid were found to be the major compounds in the analyzed mushrooms samples. Based on the results shown in Figure 2A it can be observed that the most important phenolic compounds found in *P. ostreatus* are 4-hydroxybenzoic acid and 5-feruloylquinic with the concentrations of 75.042 mg/100 g·fw and 35.040 mg/100 g·fw respectively. *p*-Hydroxybenzoic acid was also found to be the main hydroxyphenolic acid in the *P. ostreatus* ethanolic extracts analyzed by liquid chromatography (297 μg/g of extract) in the study performed by Toafiq et al. [17]. Instead, other authors didn’t identified the above mentioned phenolic acid in the extracts obtained from Korean *P. ostreatus* but they reported the presence of protocatechuic acid as well as the presence of chlorogenic acid and myricetin which were not detected in our sample [18]. 

The separation of phenolic acids and flavonol glycosides in *A. bisporus* extract is shown in Figure 2B. The highest content in the analyzed extract is represented by 5-ferulylquinic acid, 71.005 mg/100 g fw and protocatechnic acid, 46.108 mg/100 g fw values which are higher than the ones reported by Palacios et al. [15]. In the same study, the main phenolic compounds identified in *A. bisporus* were catechin, chlorogenic acid, homogentisic acid pyrogallol, and myricetin [15].

Reis et al. reported in their study the presence in *A. bisporus* of the cinnamic acid which is in good correlation with our results [4]. The analyzed extract was also characterized by a large amount of phenolic acids such as 4-ferulylquinic acid (60.458 mg/100 g fw) and 4-hydroxybenzoic acid (79.495 mg/100 g fw).

Figure 2C shows the composition of phenolic acids and flavonol glycosides in *C. cibariu* extract. It can be noticed that 5-feruloylquinic acid and 3,5-dicaffeoylquinic acid were the major ones, accounting for 55.32 mg/100 g fw and 54.20 mg/100 g fw, respectively. The 4-hydroxybenzoic acid concentration (16.159 mg/100 g fw) in *C. cibariu* exceeds the value reported by Palacios et al. and Muszynska et al. for the same mushroom variety [15,19]. It is also important to note that the content in phenolic compounds is dependent on extrinsic factors such as the geographical origin but also by the method used for the extraction of these bioactive compounds and the efficiency of extraction solvent. For instance Barros et al. found a concentration of 1.497 mg/100 g fw of cinnamic acid in *C. cibariu* extract while in our samples the concentration of the same phenolic acid was with 60% higher [20]. Moreover, differently from the Portuguese *C. cibariu,* in our sample, 3-feruloylquinic acid and 4-feruloylquinic acid were also identified, having concentrations of 9.492 mg/100 g fw and 6.314 mg/100 g fw respectively.

According to Figure 2D *B. edulis* is much richer in phenolic compounds than *A. bisporus* and *C. cibarius.* The results revealed that the *Boletus edulis* extract has the highest concentration of 4-hydroxybenzoic acid, 209.867 mg/100 g fw, this value being 2.5 times larger than the one in *A. bisporus* and by 15 times bigger than in *C. cibarius*. Similar values (240 mg/100 g fw) were reported for *B. edulis* in the study conducted by Palacios et al. [15]. Also, *B. edulis* extract is characterized by high concentrations of cinnamic acid 168.614 mg/100 g fw and catechin 145.566 mg/100 g fw, comparable with the ones found by Taofiq et al. [17].

*L. piperatus* (Figure 2E) even if it is considered a less valued mushroom than *B. edulis*, it contains 3-feruloylquinic acid, 4-feruloylquinic acid and 5-feruloylquinic acid, which were not identified in *B. edulis*. Furthermore, 3-feruloylquinic acid was found only in *L. piperatus* and *C. cibarius*, having a concentration of seven times higher in *L. piperatus*. It can be also observed that *L. piperatus* has the highest content of 4-feruloylquinic acid, 87.621 mg/100 g fw, surpassing with 45% *A. bisporus*, which has the second largest 4-feruloylquinic acid concentration (60.4 mg/100 g fw). It is worth noticing that *L. piperatus* is the only mushroom specie among the studied ones in which ferulic acid has been identified, presenting a concentration of 9.153 mg/100 g fw.

In accordance with previous studies the obtained results revealed that the five Transylvanian wild edible mushrooms can compete with other important sources of polyphenols, being rich in bioactive compounds [21].

## 3. Materials and Methods

### 3.1. Experimental Material

All the fruiting bodies of selected species (*Agaricus bisporus, Pleurotus ostreatus, Cantharellus cibarius, Boletus edulis and Lactarius piperatus*) were collected from Meseş mountain, Sălaj county, in Transylvania region, Romania. The selected mushroom species are members of the Agaricomycetes class, while: (i) *P. ostreatus* is member of Pleurotaceae family; (ii) *A. bisporus* is part of the Agaricaceae family; (iii) *C. cibarius* is from the Cantharellaceae family; (iv) *B. edulis* is member of the Boletaceae family and (v) *L. piperatus* is part of the Russulaceae family. An amount of 4 kg of fresh and cleaned fruiting bodies from each mushroom species was randomly divided, cut in thin slices and dried at 25 °C for 24 h, using a laboratory plant dryer. Afterwards, the mushroom material was ground into a fine powder using a laboratory mill, mixed to obtain a homogeneous sample and kept at 4 °C for one week, until analysed. 

### 3.2. Proximate Composition

The chemical composition of five wild edible Romanian mushrooms, including moisture, ash, total carbohydrates, total sugars, crude fat and crude protein, were determined according to AOAC procedures [22]. To obtain the moisture content, samples of the mushrooms were dried at 105 °C until constant weight. The ash content was determined by incineration at 600 ± 15 °C for 6 h. The crude protein content of the samples was estimated by the Kjeldahl method. For the calculation of crude protein in mushrooms, the nitrogen content was multiplied by a factor of 4.38 [23]. The crude fat content of the samples was determined by extracting a know weight of powdered mushroom sample (3g) with petroleum ether as a solvent, using Soxhlet apparatus [24]. The amount of total carbohydrate was calculated by difference [1]:

(1) total carbohydrate=100−(g moisture+g protein+g fat+g ash)

The total energy was calculated according to the following equations [1]:

(2) energy=4×(g protein+g carbohydrate)+9·(g lipid)[kcal]

### 3.3. Extraction of Lipids and Fatty Acid Composition

The total lipids (TLs) of dried samples powder (10 g) were extracted using a chloroform: methanol mixture (2:1, *v*/*v*, 60 mL) using a high power homogenizer (MICCRA D-9, ART Prozess-und Labortechnik, Müllheim, Germany) [25]. The mixture was filtered and the residue was washed with chloroform: methanol (2:1, *v*/*v*, 60 mL). The filtrates and washings were combined and cleaned with 0.88% aqueous potassium chloride (45 mL) followed by methanol: aqueous potassium chloride solution (1:1, *v*/*v*, 40 mL). The purified lipid layer was filtered and dried over anhydrous sodium sulphate and the solvent was removed in a rotary evaporator (Laborata 4010 Digital, Heidolph, Schwabach, Germany). The recovered oils were weighed, transferred into vials with 3 mL chloroform and stored at 18 °C for further analysis.

Fatty acid methyl esters (FAMEs) of the TLs were obtained by acid catalyzed transesterification using methanolic H_2_SO_4_ (1% *v*/*v*) [26]. Shortly, lipids (1 mg) were re-suspended in 1 mL toluene in a Pyrex tube fitted with a condenser. Two milliliters of methanolic H_2_SO_4_ were added, and the mixture was refluxed for 2 h at 80 °C. Five mL of aqueous potassium chloride solution (5% *w*/*v*) were added and the trans-methylated fatty acids extracted with hexane (2 × 5 mL). The hexane layer was washed with 4 mL of 2% potassium bicarbonate solution and dried over anhydrous sodium sulphate. Finally, the solution was filtered and the solvent was removed under reduced pressure in a rotary film evaporator. The FAMEs were analyzed by gas chromatography–mass spectrometry (GC-MS) using a Perkin Elmer Clarus 600 T GC-MS system (PerkinElmer, Inc., Shelton, CT, USA) [27]. The initial oven temperature was 140 °C, programmed by 7 °C /min to 220 °C and kept 23 min at this temperature. The injector was set at 210 °C, with a 1:24 split ratio, and 0.5 μL of sample were injected. The carrier gas was He with a flow rate of 0.8 mL/min. The EI (positive ion electron impact) mass spectra were recorded at 70 eV and a trap current of 100 μA with an ion source temperature of 150 °C. The mass scans were performed within the range 22–395 *m*/*z*. Identification of FAMEs was accomplished by comparing their retention times with those of known standards and the resulting mass spectra to those in the database (NIST MS Search 2.0). The amount of each fatty acid was expressed as percent of total fatty acid content.

### 3.4 Extraction and Analysis of Volatile Compounds

The extraction of volatile compounds was performed using the in-tube extraction technique (ITEX) as described in our previous work [28] using 0.5 g of sample. The analysis of volatile compounds was carried out on a GCMS QP-2010 (Shimadzu Scientific Instruments, Kyoto, Japan) model gas chromatograph-mass spectrometer. The volatile compounds were separated on a Zebron ZB-5ms capillary column of 50 m × 0.32 mm i.d and 0.25 µm film thickness. The carrier gas was He, 1 ml/min and the split ratio 1:20. The temperature program used for the column oven was: 40 °C (held for 6 min) rising to 50 °C at 2 °C/min, heated to 250 °C at 7 °C/min and held for 2 min. The injector, ion source and interface temperatures were set at 250 °C. The MS mode was electron impact (EI) at an ionization energy of 70 eV. The scanned mass range was 35–350 *m*/*z*.

The volatile compounds were tentatively identified using the spectra of reference compounds from NIST27 and NIST147 mass spectra libraries and verified by comparison with retention indices drawn from www.pherobase.com or www.flavornet.org (for columns with a similar stationary phase to the ZB-5ms column). The compounds were considered “tentatively identified” only in the case in which their mas spectra similarity value was above 85%. All peaks found in at least two of the three total ion chromatograms (TIC) were considered when calculating the total area of peaks (100%) and the relative areas of the volatile compounds. 

### 3.5 HPLC–ESI/MS Identification Analysis of Phenolic Compounds

Samples were analyzed on an Agilent Technologies 1200 HPLC system (Chelmsford, MA, USA) equipped with G1311A Quaternary Pump, G1322A degasser, G1329A autosampler and G1315D photo-diode array (PDA) detector. Volumes of 10 µL were injected on an Eclipse XDB C18 column (4.6 × 150 mm, 5 μm, Agilent Techologies). The mobile phase was composed of 0.1% acetic acid in bidistilled water (solvent A) and 0.1% acetic acid acetonitrile (solvent B). The flow rate was maintained at 0.5 mL/min and column temperature was maintained at 25 °C. The gradient elution system started with 5% B for 2 min. The percent of B increased linearly to 40% at 18 min and continued up to 90% B at 24 min. Between 25 and 30 min the percentage of B was 5%. PDA recorded full spectra. In-line MS data were recorded by directing the LC flow to a Quadrupole 6110 mass spectrometer (Agilent Technologies) equipped with an ESI probe. The spray voltage was set at 3000 V. Nitrogen was used as nebulizer gas and nebulizer pressure was set to 40 psi with a source temperature of 100 °C. Desolvation gas (nitrogen) was heated at 300 °C and delivered at a flow of 0.8 mL/min. Mass spectra were acquired in ESI and full scan mode in a range of 100–1000 *m*/*z*. Molecular ions and fragment ions were determined by setting the fragmentation voltage at 70 and 130 eV. Identification of flavonoid and phenolic acids was carried out based on molecular mass determination, masses and occurrence of fragments using Agilent ChemStation software and also based on the compounds elution order and literature data reported previously [15,20]. 

### 3.6. RP-HPLC-PDA Quantification Analysis of Phenolic Compounds

HPLC analysis of the samples was performed on a Shimadzu system equipped with a binary pump delivery system (model LC-20 AT Prominence), a degasser (model DGU-20 A3 Prominence), and a UV-Vis diode array detector (model SPD-M20). The separation was done using a Luna C-18 column (film thickness, 5 µm; 25 cm, 4.6 mm) (Phenomenex, Torrance, CA, USA) and the mobile phases consisted in formic acid (4.5%) in double-distilled water (solvent A) and acetonitrile (solvent B). The gradient elution system was as follows: 10% B, 0–9 min; 12% B, 9–17 min; 25% B, 17–30 min; 90% B, 30–50 min; and 10% B, 50–55 min. The flow rate was set at 0.8 mL/min, and all analyses were performed at controlled temperature 35 °C. The chromatograms were monitored at 280 nm. The phenolic acids quantification was done using a gallic acid standard curve.

### 3.7. Statistical Analysis

The analyses were performed in triplicate and the obtained results were expressed as mean ± standard deviation (SD) for each sample. The software employed for statistical analysis was Graphpad Prism 7, version 7.03 for Windows (GraphPad Software, San Diego, CA, USA).

## 4. Conclusions

The current study revealed through an in-depth characterization of chemical composition that fruiting bodies of the selected wild edible mushrooms from Romania are sources of bioactive molecules, including PUFAs and phenolic compounds. Thus, *P. ostreatus*, *A. bisporus* and *B. edulis* were riched in PUFAs, especially linoleic acid (59.88–71.41%), while *C. cibarius* and *L. piperatus* were characterized by higher percentages of MUFAs and SFAs. The phenolic profiles of studied mushrooms were different, each species being distinguished from qualitative or quantitative point of view. Hence, enven though 4-hydroxybenzoic acid, protocatechuic acid, catechin or cinnamic acids were present in all samples, their concentration varied widely. Regarding the volatile profile, aldehydes and ketones as well as the sulphur compounds in the case of *B. edulis* and *L. piperatus* were the most abundant. The main aldehydes present in all samples were 2-methylpentanal, hexanal and benzaldehyde but their concentration varied between analyzed mushrooms. Even though variations in chemical composition of the mushroom species may occur due to intrinsic and extrinsic factors, the characterization of these five wild edible mushrooms species from Transylvania can represent ground data for further studies related to the possibilities and sustainability of their use in developing new functional products and/or ingredients. 

## Figures and Tables

**Table 1:** Nutritional value of the studied wild edible mushrooms in Transylvania.

Samples	Moisture (g/100 g dw)	Total Fat (g/100 g dw)	Crude Protein (g/100 g dw)	Ash (g/100 g dw)	Carbohydrates (g/100 g dw)	Energy (kcal/100 g dw)
*P. ostreatus*	7.26 ± 0.08	1.26 ± 0.08	17.92 ± 0.18	11.11 ± 0.22	62.45 ± 0.58	332.80 ± 0.82
*A. bisporus*	5.35 ± 0.07	2.41 ± 0.01	34.68 ± 0.17	7.24 ± 0.05	50.33 ± 0.31	361.70 ± 0.44
*C. cibarius*	2.17 ± 0.02	2.17 ± 0.04	21.03 ± 0.04	9.57 ± 0.08	65.06 ± 0.11	363.9 ± 0.66
*B. edulis*	7.23 ± 0.18	1.92 ± 0.09	36.24 ± 0.12	8.38 ± 0.07	46.23 ± 0.22	347.5 ± 0.52
*L. piperatus*	14.20 ± 0.27	2.69 ± 0.05	31.81 ± 0.15	8.30 ± 0.21	43.01 ± 0.70	323.50 ± 1.67

*P. ostreatus—Pleurotus ostreatus, A. bisporus—Agaricus bisporus, C. cibarius—Cantharellus cibarius, B. edulis—Boletus edulis, L. piperatus—Lactarius piperatus*. Each value is expressed as mean ± SD (n = 3).

**Table 2:** Fatty acid composition (%) of the selected Transylvanian Mushrooms.

No	Fatty Acid	*P. ostreatus*	*A. bisporus*	*C. cibarius*	*B. edulis*	*L. piperatus*
1	(6:0)	0.02 ± 0.001	n.d.	0.02 ± 0.001	0.15 ± 0.002	0.25 ± 0.02
2	(8:0)	0.02 ± 0.001	0.01 ± 0.001	0.01 ± 0.001	0.12 ± 0.01	0.07 ± 0.005
3	(10:0)	0.06 ± 0.002	0.02 ± 0.001	0.06 ± 0.006	0.16 ± 0.003	0.17 ± 0.002
4	(12:0)	0.03 ± 0.001	0.01 ± 0.01	0.03 ± 0.002	0.07 ± 0.002	0.05 ± 0.001
5	(14:0)	0.47 ± 0.001	0.55 ± 0.005	0.13 ± 0.001	0.22 ± 0.001	0.15 ± 0.001
6	(15:0)	1.87 ± 0.01	1.17 ± 0.02	0.49 ± 0.01	0.26 ± 0.001	0.33 ± 0.02
7	(16:0)	12.40 ± 0.07	12.73 ± 0.07	5.35 ± 0.5	9.93 ± 0.01	15.11 ± 0.04
8	(16:1)n-9	0.16 ± 0.01	0.04 ± 0.002	0.13 ± 0.002	0.14 ± 0.001	0.21 ± 0.04
9	(16:1)n-7	0.47 ± 0.002	0.28 ± 0.001	0.24 ± 0.004	0.96 ± 0.02	0.07 ± 0.001
10	(17:0)	0.19 ± 0.02	0.71 ± 0.01	0.06 ± 0.002	0.04 ± 0.001	0.11 ± 0.001
11	(18:0)	2.18 ± 0.01	5.00 ± 0.5	43.23 ± 0.04	3.32 ± 0.5	9.01 ± 0.4
12	(18:1)n-9	15.96 ± 0.04	1.80 ± 0.02	36.29 ± 0.01	22.06 ± 0.01	9.84 ± 0.6
13	(18:1)n-7	0.12 ± 0.005	0.24 ± 0.001	0.12 ± 0.001	0.96 ± 0.002	18.58 ± 0.02
14	(18:2)n-6	63.68 ± 0.001	71.41 ± 0.04	11.72 ± 0.04	59.88 ± 0.04	44.51 ± 0.06
15	(18:3)n-3	0.12 ± 0.001	0.10 ± 0.002	0.03 ± 0.001	0.05 ± 0.002	0.12 ± 0.001
16	(20:0)	0.20 ± 0.003	2.55 ± 0.01	1.14 ± 0.03	0.23 ± 0.02	0.26 ± 0.004
17	(20:1)n-9	0.16 ± 0.01	0.12 ± 0.001	n.d.	0.21 ± 0.001	0.74 ± 0.001
18	(20:2)n-6	0.24 ± 0.006	0.25 ± 0.001	0.03 ± 0.002	0.22 ± 0.001	n.d.
19	(21:0)	0.04 ± 0.03	0.38 ± 0.004	n.d.	n.d.	n.d.
20	(22:0)	0.64 ± 0.02	1.60 ± 0.02	0.39 ± 0.004	0.18 ± 0.002	n.d.
21	(22:1)n-9	0.18 ± 0.01	0.04 ± 0.006	n.d.	0.12 ± 0.002	n.d.
22	(23:0)	0.06 ± 0.004	0.20 ± 0.001	n.d.	0.07 ± 0.004	0.15 ± 0.001
23	(24:0)	0.21 ± 0.03	0.77 ± 0.01	0.52 ± 0.005	0.52 ± 0.001	0.25 ± 0.001
24	(24:1)	0.53 ± 0.02	0.02 ± 0.001	n.d.	0.14 ± 0.01	n.d.
	Total SFA	18.38 ± 0.04	25.69 ± 0.01	51.43 ± 0.03	15.26 ± 0.07	25.93 ± 0.06
	Total MUFA	17.58 ± 0.01	2.55 ± 0.05	36.78 ± 0.01	24.59 ± 0.01	29.44 ± 0.01
	Total PUFA	64.04 ± 0.03	71.75 ± 0.03	11.79 ± 0.01	60.14 ± 0.04	44.63 ± 0.06
	PUFAs/SFAs	3.49 ± 0.002	2.79 ± 0.03	0.23 ± 0.001	3.94 ± 0.02	1.72 ± 0.002

*P. ostreatus—Pleurotus ostreatus, A. bisporus—Agaricus bisporus, C. cibarius—Cantharellus cibarius, B. edulis—Boletus edulis, L. piperatus—Lactarius piperatus*, n.d.—not detected; SFA—saturated fatty acids; MUFA—monounsaturated fatty acids; PUFA—polyunsaturated fatty acids; tr-trace; Caproic, (6:0); Caprylic, (8:0); Capric, (10:0); Lauric, (12:0); Myristic, (14:0); Pentadecanoic, (15:0); Palmitic, (16:0); *Z*-7-Hexadecenoic, [C16:1(n-9)]; Palmitoleic, [16:1(n-7)]; Margaric, (C17:0); Stearic, (18:0); Oleic, [18:1(n-9)]; Vaccenic, [18:1(n-7)]; Linoleic, [18:2(*Z*,*Z*) (n-6)]; Linoleic, [18:2(*E*,*E*) (n-6)]; α-Linolenic, [18:3(n-3)]; Arachidic, (20:0); 11-Eicosenoic, [20:1(n-9)]; Eicosadienoic, [20:2 (n-6)]; Heneicosanoic, (21:0); Behenic, (22:0); Erucic, [22:1(n-9)]; Tricosanoic, (23:0); Lignoceric, (24:0); Nervonic, [24:1(n-9)]; Azelaic, AzA. Each value is expressed as mean ± SD (n = 3). The retention times (min) for the fatty acid are specified in the chromatogram presented in Figure 1.

**Table 3:** Mean relative peak areas (expressed as % from total peak areas) and standard deviations of volatile compounds from mushroom samples analysed by HS-ITEX/GC-MS technique.

No.	Volatile Compound	Odour Perception	*P. ostreatus*	*A. bisporus*	*C. cibarius*	*B. edulis*	*L. piperatus*
	***Alcohols***						
1.	2-Methyl-1-butanol	Malty, alcoholic, fruity, whiskey, burnt	1.39 ± 0.33	n.d.	n.d.	n.d.	1.26 ± 0.07
2.	1-Pentanol	Sweet, balsamic, fruity	n.d.	n.d.	1.3 ± 0.12	n.d.	n.d.
3.	1-Hexanol	resin, flower, green	n.d.	n.d.	0.86 ± 0.30	n.d.	n.d.
4.	1-Octen-3-ol	mushroom	n.d.	n.d.	1.36 ± 0.06	4.87 ± 0.07	n.d.
5.	1-Dodecanol	waxy, fatty, honey	n.d.	2.09 ± 0.46	0.66 ± 0.14	7.09 ± 0.23	n.d.
	Total		1.39 ± 0.33	2.09 ± 0.46	4.18 ± 0.62	11.96 ± 0.30	1.26 ± 0.07
	***Aldehydes***						
1.	2-Methylpentanal	ethereal fruity green	19.26 ± 0.35	7.56 ± 0.16	4.45 ± 0.10	4.45 ± 0.23	8.83 ± 0.48
2.	Hexanal	Green, grass, fat	8.45 ± 0.25	19.12 ± 0.31	55.48 ± 0.29	11.13 ± 0.03	5.04 ± 0.68
3.	Heptanal	Fat, citrus, rancid	n.d.	2.06 ± 0.16	1.82 ± 0.02	4.01 ± 0.17	n.d.
4.	Benzaldehyde	Almond, burnt sugar	43.36 ± 1.28	36.72 ± 0.31	6.12 ± 0.81	13.12 ± 0.29	6.12 ± 0.81
5.	Octanal	Fat, soap, lemon, green	1.43 ± 0.21	2.98 ± 0.22	0.99 ± 0.07	4.13 ± 0.31	1.88 ± 0.23
6.	Benzeneacetaldehyde	Harsh, green, honey, cocoa	n.d.	1.28 ± 0.10	n.d.	n.d.	n.d.
7.	(*E*)-2-Octenal	Green, nut, fat	n.d.	n.d.	0.66 ± 0.07	n.d.	n.d.
8.	Nonanal	Fat, citrus, green	2.34 ± 0.09	2.33 ± 0.33	0.71 ± 0.11	3.34 ± 0.02	2.33 ± 0.39
9.	Decanal	Soap, orange peel, tallow	n.d.	n.d.	n.d.	1.09 ± 0.07	n.d.
	Total		74.84 ± 2.18	72.05 ± 1.59	70.23 ± 1.47	41.27±1.12	24.20 ± 2.59
	***Ketones***		n.d.	n.d.			
1.	2-Hexanone	fruity, fungal, meaty, buttery	n.d.	n.d.	0.6 ± 0.06	n.d.	
2.	2,3-Dimethylcyclopentanone		n.d.	n.d.	n.d.	n.d.	3.85 ± 0.18
3.	2-Heptanone	Sulfur, pungent, green, fruity	12.61 ± 0.59	n.d.	3.83 ± 0.09	n.d.	2.19 ± 0.03
4.	1-Octen-3-one	herbal, mushroom, earthy, musty	n.d.	n.d.	0.56 ± 0.06	n.d.	n.d.
5.	3-Octanone	Musty, mushroom, herbal	n.d.	n.d.	0.13 ± 0.02	6.46 ± 0.51	n.d.
6.	Acetophenone	Sweet, flower, almond	n.d.	1.72 ± 0.08	0.42 ± 0.09	2.79 ± 0.42	0.65 ± 0.04
7.	Benzophenone	balsam rose metallic powdery geranium	n.d.	3.09 ± 0.12	0.92 ± 0.18	7.81 ± 0.76	n.d.
	Total		12.61 ± 0.59	4.81 ± 0.20	6.46 ± 0.50	17.06 ± 1.69	6.69 ± 0.25
	***Terpenoids***						
1.	α-Pinene	pine, turpentine	n.d.	n.d.	0.40 ± 0.08	n.d.	n.d.
2.	β-Phellandrene	mint, terpentine	n.d.	1.42 ± 0.03	0.53 ± 0.05	n.d.	n.d.
3.	β-Pinene	pine, resin, turpentine	n.d.	1.71 ± 0.29	1.16 ± 0.03	n.d.	1.52 ± 0.27
4.	Β-Myrcene	balsamic, must, spice	n.d.	n.d.	n.d.	4.56 ± 0.55	n.d.
5.	D-Limonene	citrus, mint, fruity	n.d.	4.55 ± 0.61	1.05 ± 0.38	n.d.	3.07 ± 0.12
	Total		n.d.	7.68 ± 0.93	3.14 ± 0.54	4.56 ± 0.55	4.59 ± 0.39
	***Sulphur compounds***						
1.	Methyl disulfide	onion, cabbage, sulfurous, vegetable	n.d.	n.d.	0.58 ± 0.10	11.56 ± 0.48	1.31 ± 0.54
2.	Methyl 2-propenyl disulfide	alliaceous, garlic, green, onion	n.d.	n.d.	2.15 ± 0.38	n.d.	22.19 ± 2.05
3.	Dimethyl trisulfide	sulfurous, cooked onion, savory, meaty	n.d.	n.d.	n.d.	5.59 ± 0.29	n.d.
4.	Diallyl disulphide	alliaceous, onion, garlic, metallic	n.d.	n.d.	2.7 ± 0.25	n.d.	27.89 ± 1.21
	Total		n.d.	n.d.	5.43 ± 0.73	17.15 ± 0.77	51.39 ± 3.80
	***Acids***						
1.	Nonanoic acid	green, fat	n.d.	n.d.	n.d.	n.d.	n.d.
2.	*n*-Decanoic acid	fat	n.d.	n.d.	0.54 ± 0.18	2.96 ± 0.45	n.d.
3.	Dodecanoic acid	fatty, waxy	5.14 ± 0.34	6.53 ± 0.27	1.99 ± 0.06	5.07 ± 0.44	6.86 ± 1.17
	Total		5.14 ± 0.34	6.53 ± 0.27	2.53 ± 0.24	8.03 ± 0.89	6.86 ± 1.17
	***Others***						
1	2-*n*-Pentylfuran	fruity, green, earthy, beany	6.05 ± 0.37	6.88 ± 0.36	11.29 ± 0.4	n.d.	5.05 ± 0.57
	Total		6.05 ± 0.37	6.88 ± 0.36	11.29 ± 0.4	n.d.	5.05 ± 0.57

*P. ostreatus—Pleurotus ostreatus, A. bisporus—Agaricus bisporus, C. cibarius—Cantharellus cibarius, B. edulis—Boletus edulis, L. piperatus—Lactarius piperatus*; n.d.—not detected. Each value is expressed as mean ± SD (n = 3).

**Table 4:** The phenolic content in analyzed mushrooms extracts determined by HPLC–DAD and expressed as mg gallic acid equivalents per 100 g·fw (fresh weight).

Peak No.	Compound	Phenolic Compound in Analyzed Mushrooms Extracts (mg/100 g fw)				
		*P. ostreatus*	*A. bisporus*	*C. cibarius*	*B. edulis*	*L. piperatus*
1	4-Hydroxybenzoic acid	75.04 ± 0.20	79.50 ± 0.02	16.16 ± 0.12	209.87 ± 0.35	42.93 ± 0.22
2	2,4-Dihydroxybenzoic acid	11.84 ± 0.20	19.62 ± 0.06	4.96 ± 0.02	69.13 ± 0.15	n.d.
3	4-Hydroxyphenylacetic acid	4.02 ± 0.05	5.06 ± 0.005	1.60 ± 0.01	25.30 ± 0.28	7.38 ± 0.03
4	Protocatechuic acid	17.28 ± 0.6	46.11 ± 0.05	5.17 ± 0.02	43.58 ± 0.25	5.48 ± 0.01
5	Catechin	14.86 ± 0.10	31.29 ± 0.02	2.43±0.06	145.57 ± 0.40	6.47 ± 0.05
6	Gallocatechin	5.04 ± 0.15	5.27 ± 0.01	1.03±0.02	26.63 ± 0.25	10.95 ± 0.2
7	*p*-Coumaric acid	n.d.	n.d.	1.47±0.01	23.11 ± 0.20	5.19 ± 0.06
8	Ferulic acid	n.d.	n.d.	n.d.	n.d.	9.15 ± 0.03
9	Sinapic acid	n.d.	n.d.	n.d.	27.38 ± 0.08	8.66 ± 0.01
10	*o*-Coumaric acid	3.63 ± 0.20	n.d.	n.d.	11.42 ± 0.06	6.37 ± 0.24
11	Cinnamic acid	10.09 ± 0.15	14.36 ± 0.02	2.38 ± 0.01	168.61 ± 0.45	14.54 ± 0.15
12	3-Feruloylquinic acid	n.d.	n.d.	9.49 ± 0.08	n.d.	66.73 ± 0.40
13	4-Feruloylquinic acid	n.d.	60.46 ± 0.06	6.31 ± 0.06	n.d.	87.62 ± 0.35
14	5-Feruloylquinic acid	35.04 ± 0.08	71.01 ± 0.04	55.33 ± 0.25	n.d.	38.19 ± 0.10
15	3,5 Dicaffeoylquinic acid	14.60 ± 0.10	14.00 ± 0.11	54.21 ± 0.13	31.55 ± 0.45	61.14 ± 0.30

n.d.—not detected. Each value is expressed as mean ± SD (n = 3).
